# Risk of SARS-CoV-2 infection in healthcare workers with inflammatory bowel disease: a case-control study

**DOI:** 10.1016/j.infpip.2022.100267

**Published:** 2022-12-30

**Authors:** Aurelien Amiot, Anne Bourrier, Jean-Marc Gornet, Olivier Dewit, Stephane Nancey, Romain Altwegg, Vered Abitbol, David Laharie, Catherine Reenaers, Charlotte Gagnière, Anthony Buisson, Maria Nachury, Stephanie Viennot, Lucine Vuitton, Carmen Stefanescu, Philippe Marteau, Guillaume Bouguen, Philippe Seksik, on behalf of the GETAID-INFOPRO study group

**Affiliations:** a Department of Gastroenterology, Bicêtre Hospital, AP-HP, Paris Est Creteil University, Le Kremlin Bicêtre, France; b Saint Antoine Hospital, Gastroenterology Unit, Centre de Recherche Saint-Antoine, Sorbonne Université, INSERM 75012, Assistance Publique-Hôpitaux de Paris, France; c Department of Gastroenterology, Saint Louis University Hospital, AP-HP, Paris, France; d Department of HepatoGastroenterology, Catholic University of Leuven, University Hospital of Saint-Luc, Brussels, Belgium; e Department of Gastroenterology, Hospices Civils de Lyon and Claude Bernard Lyon 1 University, Pierre-Benite, France; f Department of Hepatogastroenterology, Saint-Eloi Hospital, Montpellier, France; g Department of Gastroenterology, Cochin University Hospital, University Paris 5 Descartes, Paris, France; h Department of Hepato-Gastroenterology, University Hospital of Bordeaux, Hôpital Haut-Lévêque, Bordeaux, France; i Department of Gastroenterology, University Hospital of Liège, Liège, Belgium; j Department of Gastroenterology, Hôpitaux Universitaires Henri Mondor, AP-HP, Créteil, France; k Department of Hepato-Gastroenterology, University Hospital Estaing of Clermont-Ferrand, Université d'Auvergne, Clermont-Ferrand, France; l Department of Gastroenterology, Huriez University Hospital, Université Lille Nord de France, Lille, France; m Department of Gastroenterology, Besançon University Hospital, Besançon, France; n Department of Gastroenterology, Caen University Hospital, F-14000, Caen, France; o Department of Gastroenterology, IBD and Nutrition Support, Beaujon Hospital, University Paris 7 Denis Diderot, Clichy, France; p Sorbonne Université-APHP, Tenon Hospital, Paris, France; q Department of Gastroenterology, Pontchaillou Hospital and Rennes University, Rennes, France

**Keywords:** Crohn's disease, Ulcerative colitis, Inflammatory bowel disease, Healthcare worker, SARS-CoV-2, COVID-19, Novel coronavirus disease, UC, ulcerative colitis, IBD, inflammatory bowel disease, TNF, tumor necrosis factor-alpha, CRP, C-reactive protein, SARS-CoV-2, severe acute respiratory syndrome coronavirus 2

## Abstract

**Background:**

Whether healthcare workers with inflammatory bowel disease (IBD) are at increased risk of Novel coronavirus disease (COVID-19) due to occupational exposure is unknown.

**Aim:**

To assess the risk of COVID-19 in healthcare workers with IBD.

**Methods:**

A case control study enrolled 326 healthcare workers with IBD from 17 GETAID centres and matched non-healthcare workers with IBD controls (1:1) for gender, age, disease subtype and year of diagnosis. The study period was year 2020 during the COVID-19 outbreak.

**Results:**

In total, 59 COVID-19 were recorded among cases (n = 32) and controls (n = 27), including 2 severe COVID-19 (requiring hospitalization, mechanic ventilation) but no death. No difference was observed between healthcare workers and controls regarding the overall incidence rates of COVID-19 4.9 ± 2.2 *vs*. 3.8 ± 1.9 per 100 patient-semesters, *P* = 0.34) and the overall incidence rates of severe COVID-19 (0.6 ± 7.8 *vs*. 0.3 ± 5.5 per 100 patient-semesters, *P* = 0.42). In multivariate analysis in the entire study population, COVID-19 was associated with patients with body mass index > 30 kg/m^2^ (HR = 2.48, 95%CI [1.13–5.44], *P* = 0.02).

**Conclusion:**

Healthcare workers with IBD do not have an increased risk of COVID-19 compared with other patients with IBD.

## Introduction

Coronavirus disease 2019 (COVID-19) is a respiratory illness caused by severe acute respiratory syndrome coronavirus 2 (SARS-CoV-2) [1]. SARS-CoV-2 has been responsible of a massive outbreak since early 2020 and is still a daily concern for worldwide healthcare systems [2].

The risk of COVID-19 or COVID-19-related mortality in patients with IBD have been widely evaluated in many studies and a recent meta-analysis of 14 studies which included 50,706 patients with IBD [3]. The prevalence of COVID-19 in patients with IBD was low and accounted for 1% of patients through October 2020. Whereas it had been presumed that patients who are immunosuppressed would be at higher risk for COVID-19 and severe COVID-19, only the use of steroids seems to impact the risk of COVID-19 [3,4].

Healthcare workers are exposed to a substantial risk for acquiring COVID-19 due to daily and close contacts with infected patients and asymptomatic carriers of SARS-CoV-2. In a recent systematic review with meta-analysis, a total of 97 studies, reported a prevalence of 11% [7%-15%] in healthcare workers and severe COVID-19 in 5% [3%–8%] [5]. In a retrospective case-control study, we showed that healthcare workers with IBD did not have an increased risk of severe infection compared with other patients with IBD, except for tuberculosis [6].

We thus conducted a multicenter case-control study in a real-life setting aiming to assess the incidence rate of COVID-19 in healthcare workers with IBD compared with other non-healthcare worker patients with IBD, and to identify the predictors of COVID-19.

## Materials and methods

### Study population

The present study was a follow-up extension of a retrospective observational multicenter case-control study conducted in 17 French and Belgian academic centers affiliated with the Groupe d'Etude Thérapeutique des Affections Inflammatoires du tube Digestif (GETAID). In this study, 482 patients with IBD who were healthcare workers and 482 controls patients were included from the MICISTA registry, a tertiary monocentric clinical database of all consecutive patients with IBD at Saint-Antoine Hospital (Paris, France) [7,8]. Patients were identified from personal databases and/or a standardized hospital inpatient diagnosis datasets. Occupational status of patients with IBD was collected in IBD databases upstream of this study. From January 2021 to October 2021, investigators were asked to report patients previously included in their center who were still followed up until December 31^st^ 2020.

The protocol was approved by the Henri Mondor Ethics Comittee/Institutional Review Board (N°0011558-2020-070) and the Commission Nationale de l'Informatique et des Libertés (CNIL N°916056). All authors had access to the study data and reviewed and approved the final manuscript.

### Data collection

Previously collected data were retrieved and updated until December 31^st^ 2020. The recorded data included a detailed account of the IBD diagnosis and history, smoking status, IBD phenotype according to the Montreal classification, medical and surgical treatment history and any serious infection history. For each of the patients, year 2020 was divided into semesters. For each semester, which was independently analyzed, the occurrence of COVID-19 and severe COVID-19, smoking status, physician global assessment of IBD activity (active or not), weight and immunosuppressive therapy (e.g., steroids, aminosalicylates, thiopurines, methotrexate, anti-TNF therapy, ustekinumab, anti-integrin therapy and tofacitinib) were assessed [9]. A semester was considered as a treatment semester if the patient received steroids, immunomodulator and/or anti-integrine therapy during at least 3 months within the studied semester.

### Case-control study

**Figure 1:**
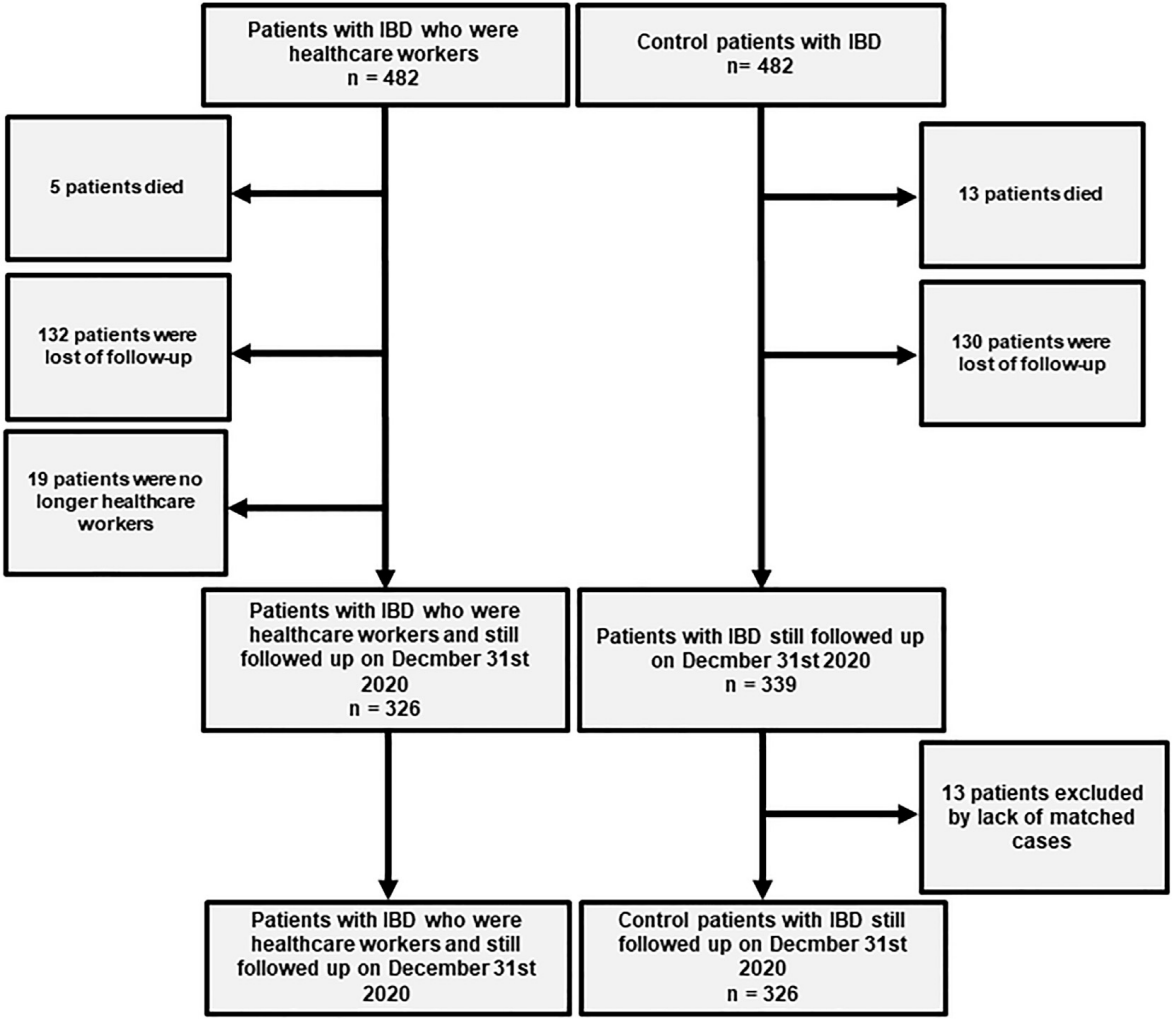
Flow-chart of the study.

In the previous study, controls were selected randomly within the MICISTA registry to match to the healthcare worker cases (1:1). MICISTA is an electronic database of the gastroenterology department of Saint-Antoine Hospital. All patients seen in the institution from 1994 are included in the database. Data regarding medical and IBD history and follow-up are prospectively coded in the system. Case-control matching was based on gender, birth year (±2.5 years), type of IBD and IBD diagnosis calendar (±2.5 years). After exclusion of 156 cases and 143 controls (Figure 1), 221 case-control couples were still available. One-hundred and five cases were rematched with 105 controls without redundancy. In total, 326 cases and 326 controls (one control for one case) were included in the present study.

### Outcomes

The main outcome measure was to compare the overall incidence of COVID-19, defined as symptomatic SARS-CoV-2 infection with a positive reverse transcription polymerase chain reaction (RT-PCR) test for SARS-CoV-2 on nasopharyngeal swab and/or a positive serological test for SARS-CoV-2 without prior vaccination. Secondary outcome was the overall incidence of severe COVID-19, defined as any COVID-19 requiring hospitalization, intensive care unit stay or death, and the impact of patients' characteristics, occupational status and ongoing treatment on the risk of COVID-19 and severe COVID-19. The rates of overall COVID-19 and severe COVID-19 were expressed for 100 patient-semester.

### Statistical analysis

Continuous data were expressed as means ± standard deviations or medians (interquartile range) whereas nominal and ordinal data were expressed as numbers and percentages. Continuous data were compared using the Chi-square test or the Fischer's exact test whenever appropriate. Parametric data were compared using the Mann-Whitney tests and Wilcoxon's matched-pair signed-rank test as appropriate. COVID-19 -free survival was calculated using the Kaplan–Meier method. To determine risk factors of COVID-19, a multivariate analyses using binary logistic regression models were performed and adjusted according to the results of univariate analysis with an ascending stepwise procedure using the Wald test. Quantitative values were converted to qualitative values using the dichotomy from the median value in two distinct groups of equal size. Variables with *P* < 0.10 in the univariate analysis were considered to be potential adjustment variables for the multivariate analysis. All analyses were two-tailed, and *P* values less than 0.05 were considered significant. Statistical evaluations were performed using SPSS statistical software (SPSS Inc., v23, Chicago, IL, USA).

## Results

### Study population

In total, 326 healthcare workers with IBD were included. The healthcare workers group included 82 (25.2%) physicians, 105 (32.2%) nurses, 41 (12.5%) nurses' aides and 98 (30.1%) other healthcare professionals (Table S1). There were 87 (26.7%) males, with a mean age of 25.9 ± 10.2 years. Types of IBD comprised 227 (69.6%) patients with Crohn's disease and 99 (30.4%) with ulcerative colitis or IBD unclassified. Patient demographic data, baseline diseases characteristics and medication history are listed in Table I.

**Table I:** Demographic and disease and medication characteristics of 652 patients with inflammatory bowel disease including 326 healthcare workers

Characteristic	Healthcare professionals (n = 326)	Controls (n = 326)	p
Age at diagnosis, years	25.9 ± 10.2	25.5 ± 10.4	0.21
Male gender, no (%)	87 (26.7%)	87 (26.7%)	1.00
BMI, kg/m^2^	23.6 ± 5.0	24.0 ± 9.4	0.50
Active smoking, no (%)	30/322 (9.3%)	47 (14.4%)	0.05
Follow-up period, years	17.8 ± 9.3	18.2 ± 9.1	0.23
Extra-intestinal manifestation, no (%)	21/301 (7.0%)	40 (12.3%)	0.03
Professional inactivity during lockdown periods, no (%)	80 (24.5%)	319 (97.9%)	<0.001
History of serious infection, no (%)	40 (12.3%)	38 (11.7%)	0.90
Age at diagnosis, no (%)			
A1: ≤16 years	25 (7.7%)	57 (17.5%)	<0.001
A2: 17–40 years	279 (85.6%)	233 (71.5%)	<0.001
A3: > 40 years	22 (6.7%)	36 (11.0%)	0.07
Crohn's disease, no (%)	227 (69.6%)	227 (69.6%)	1.00
Disease location, no (%)			
Ileal	81/226 (35.8%)	92/227 (40.5%)	0.33
Colonic	53/226 (23.5%)	51/227 (22.5%)	0.82
Ileocolonic	83/226 (36.7%)	84/227 (37.0%)	1.00
Upper GI tract	22/226 (9.7%)	27/227 (11.9%)	0.55
Disease phenotype, no (%)			
Non structuring – Non penetrating	107/210 (51.0%)	122/227 (53.7%)	0.57
Stricturing	53/210 (25.2%)	41/227 (18.1%)	0.08
Penetrating	50/210 (23.8%	64/227 (28.2%)	0.33
Perianal disease, no (%)	77 (23.6%)	75 (23.0%)	0.93
Physician global assessment of IBD activity, no (%)	34/324 (10.5%)	29 (8.9%)	0.51
Ulcerative colitis and IBDU, no (%)	99 (30.4%)	99 (30.4%)	1.00
Proctitis	11 (11.1%)	11/97 (11.3%)	1.00
Left-sided colitis	30 (30.3%)	36/97 (37.1%)	0.37
Pancolitis	58 (58.6%)	50/97 (51.5%)	0.39
History of intestinal resection, no (%)	97 (32.0%)	111 (34.0%)	0.61
Current treatment, no (%)			
None	57 (17.5%)	53 (16.3%)	0.75
Aminosalicylates	53 (16.3%)	63 (19.3%)	0.36
Immunosuppressant alone	26 (8.0%)	29 (8.9%)	0.78
Anti-TNF monotherapy	105 (32.2%)	110 (33.7%)	0.74
Anti-TNF combotherapy	24 (7.4%)	26 (8.0%)	0.88
vedolizumab	17 (5.2%)	11 (3.4%)	0.33
ustekinumab	42 (12.9%)	31 (9.5%)	0.21
tofacitinib	2 (0.6%)	2 (0.6%)	1.00

BMI: body mass index; COVID-19: Novel coronavirus disease; GI: gastrointestinal; IBD: inflammatory bowel disease; IBDU: inflammatory bowel disease undetermined.

Variables are presented as n (%), mean ± standard deviation or median (interquartile range).

*P* values are based on a two-sided chi-square test for all categorical variables and on Wilcoxon's matched-pair signed-rank test for all quantitative variables.

The control group included 326 non-healthcare worker patients with IBD. Patient demographic data, baseline diseases characteristics and medication history of the control group are also listed in Table I. The two groups were well balanced except for a slight difference in the age at diagnosis distribution according to the Vienna classification but without significant difference on the mean age at diagnosis (25.9 ± 10.2 vs. 25.5 ± 10.4, *P* = 0.21). Healthcare workers were less likely to smoke (9.3% vs. 14.4%, *P* = 0.05) and were also less frequently confined at home during the lockdown periods (16.6% *vs*. 14.4%, *P* < 0.001) which is consistent with their occupational status.

### Incidental cases of COVID-19

During year 2020, we collected 57 overall incidental COVID-19 event (8.7%) in 32 case (9.8%) and 25 control (7.7%) patients accounting for an incidence rate of 4.4 ± 2.2 overall COVID-19 per 100 patient-semesters (Table II). No difference was noted between the healthcare workers and the control group regarding the incidence rate of COVID-19 (4.9 ± 2.2 *vs*. 3.8 ± 1.9 per 100 patient-semesters, *P* = 0.34) (Table II).

**Table II:** Incidence rates of COVID-19 in 652 patients with inflammatory bowel disease according to healthcare worker status

Characteristic	Healthcare personal (n = 326)	Non-healthcare personal (n = 326)	Overall study population (n = 652)	*P* Value
SARS-CoV-2 infection	32 events 4.9 ± 2.2	25 events 3.8 ± 1.9	57 events 4.4 ± 2.2	0.34
Severe SARS-CoV-2 infection	**4 events** **0.6** ± 7.8	**2 events** **0.03** ± 5.5	**6 events** **0.5** ± 6.8	**0.42**

Incidence rates are expressed as events per 100 patient-semesters.

COVID-19: Novel coronavirus disease.

**Figure 2:**
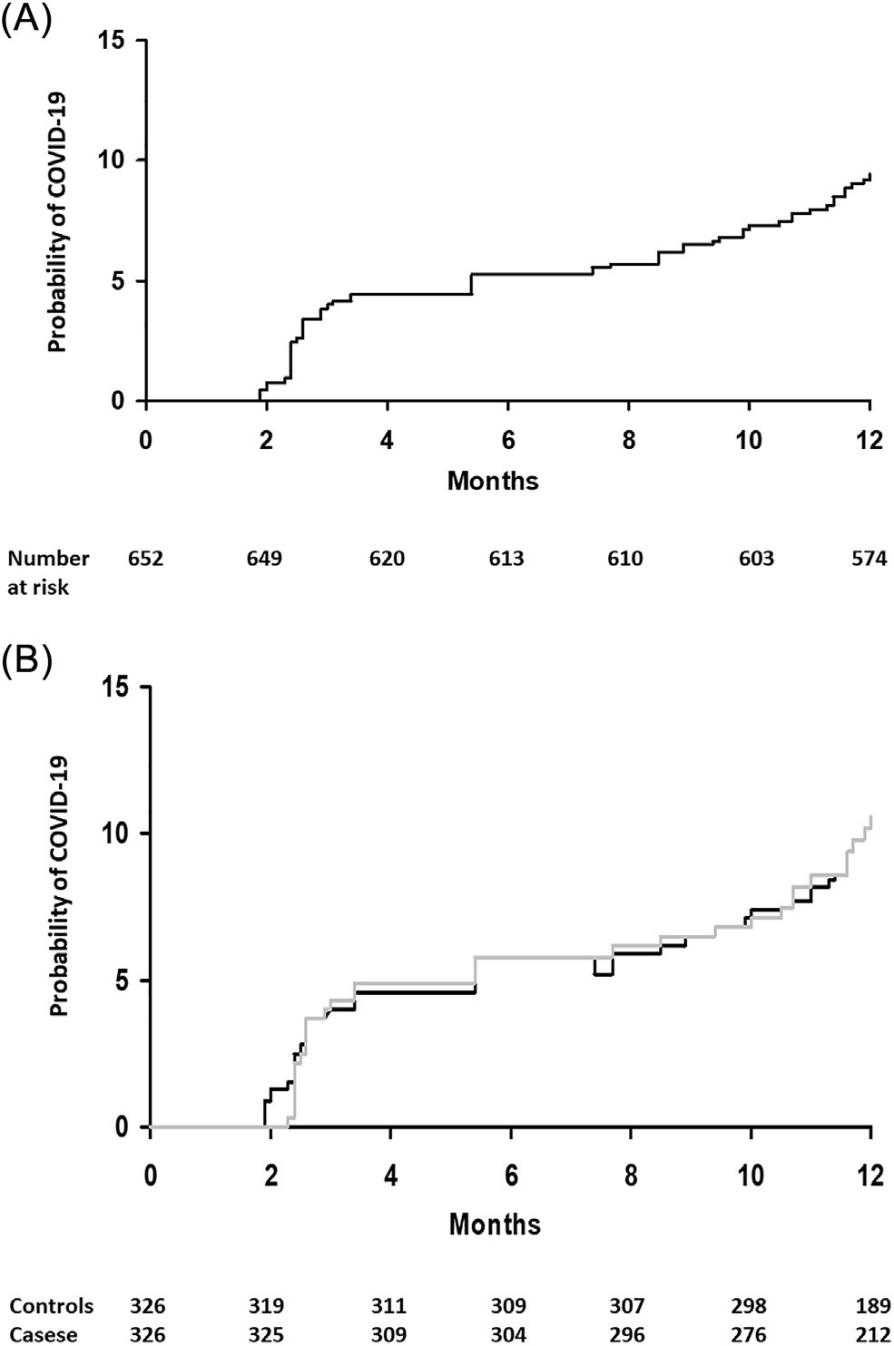
Kaplan–Meier curves of 652 patients with inflammatory bowel disease assessing the occurrence of COVID-19 in the whole cohort (A) and according to occupational status (B).

In total, we collected six severe COVID-19 in four case and two control patients accounting for an incidence rate of 0.5 ± 6.8 overall severe COVID-19 per 100 patient-semesters (Table II). No deaths were noted in healthcare workers and the control group. One patient required admission in intensive care unit and subsequently recovered. No difference was noted between the healthcare workers and the control group regarding the incidence rate of severe COVID-19 (0.6 ± 7.8 *vs*. 0.3 ± 5.5 per 100 patient-semesters, *P* = 0.42) (Table II). The probability of developing COVID-19 in the whole cohort were 3.7%, 4.9%, 6.2% and 9.1% at 3, 6, 9 and 12 months, respectively (Figure 2).

### Predictors of COVID-19

Predictors of overall severe infection were assessed in the entire study population including cases and controls. In univariate analysis, patients with BMI > 25 kg/m^2^ (*P* = 0.05) and > 30 kg/m^2^ (*P* = 0.03), nurses' aides (*P* = 0.02), Crohn's disease of the upper GI tract (*P* = 0.003) and with proctitis E1 (*P* = 0.06) (Table III). In multivariate analysis stratified on smoking habits, patients with BMI > 30 kg/m^2^ (HR = 2.48, 95%CI [1.13–5.44], *P* = 0.02) were more likely to develop COVID-19.

**Table III:** Demographic and disease and medication characteristics of 652 patients with inflammatory bowel disease according to incidental COVID-19

Characteristic	Absence of COVID-19 (n = 595)	COVID-19 (n = 57)	P
**Age, years**	43.6 ± 12.1	44.5 ± 11.6	0.60
Age > 50 years	170 (28.6%)	21 (36.8%)	0.22
Age > 60 years	73 (12.3%)	6 (10.5%)	0.83
**Male gender, no (%)**	162 (27.2%)	12 (21.1%)	0.35
BMI, kg/m2	23.8 ± 7.3	24.2 ± 6.1	0.54
BMI > 25 kg/m^2^	157/529 (29.7%)	22/50 (44.0%)	0.05
BMI > 30 kg/m^2^	43/529 (8.1%)	9/50 (18.0%)	0.03
**Active smoking, no (%)**	69/592 (11.7%)	8/56 (14.3%)	0.52
**Follow-up period, years**	17.8 ± 9.0	19.8 ± 10.7	0.11
**Extra-intestinal manifestation, no (%)**	57 (9.6%)	7 (12.3%)	0.49
**Healthcare worker, no (%)**	294 (49.6%)	32 (54.2%)	0.59
Physician	75 (12.6%)	7 (12.3%)	1.00
Nurse	96 (16.1%)	9 (15.8%)	1.00
Nurse's aid	33 (5.5%)	8 (14.0%)	0.02
Other	90 (15.1%)	8 (14.0%)	1.00
**Professional inactivity during lockdown periods, no (%)**	364 (61.2%)	35 (61.4%)	1.00
**History of serious infection, no (%)**	74 (12.6%)	3 (5.3%)	0.13
**Age at diagnosis, no (%)**			
A1: ≤16 years	72 (12.1%)	10 (17.5%)	0.29
A2: 17–40 years	470 (79.0%)	42 (73.7%)	0.40
A3: > 40 years	53 (8.9%)	5 (8.8%)	1.00
**Crohn's disease, no (%)**	417 (70.1%)	37 (64.9%)	0.45
Disease location, no (%)			
Ileal	157/416 (37.7%)	16 (43.2%)	0.60
Colonic	98/416 (23.6%)	6 (16.2%)	0.42
Ileocolonic	154/416 (37.1%)	13 (35.1%)	0.86
Upper GI tract	39/416 (9.4%)	10 (27.0%)	0.003
Disease phenotype, no (%)			
Non structuring – Non penetrating	212/401 (52.9%)	18/36 (47.2%)	0.60
Stricturing	83/401 (20.7%)	11/36 (30.6%)	0.20
Penetrating	106/401 (26.4%)	8/36 (22.2%)	0.69
Perianal disease, no (%)	139 (23.4%)	13 (22.8%)	1.00
**Physician global assessment of IBD activity, no (%)**	60/593 (10.1%)	3 (5.3%)	0.35
**Ulcerative colitis and IBDU, no (%)**	177 (29.8%)	21 (35.6%)	0.45
Proctitis	17/176 (9.7%)	5 (25.0%)	0.06
Left-sided colitis	60/176 (34.1%)	6 (30.0%)	0.81
Pancolitis	99/176 (56.3%)	9 (45.0%)	0.35
**History of intestinal resection, no (%)**	188/576 (32.6%)	20/53 (37.7%)	0.45
**Current treatment, no (%)**			
None	100 (16.8%)	10 (17.5%)	0.85
Aminosalicylates	104 (17.5%)	12 (21.1%)	0.47
Immunosuppressant alone	51 (8.6%)	4 (7.0%)	1.00
Anti-TNF monotherapy	198 (33.3%)	17 (29.8%)	0.66
Anti-TNF combotherapy	47 (7.9%)	3 (5.3%)	0.61
vedolizumab	24 (4.0%)	4 (7.0%)	0.30
ustekinumab	66 (11.1%)	7 (12.3%)	0.83
tofacitinib	4 (0.7%)	0	1.00

BMI: body mass index; COVID-19: Novel coronavirus disease; GI: gastrointestinal; IBD: inflammatory bowel disease; IBDU: inflammatory bowel disease undetermined.

Variables are presented as n (%), mean ± standard deviation or median (interquartile range).

*P* values are based on a two-sided chi-square test for all categorical variables and on Wilcoxon's matched-pair signed-rank test for all quantitative variables.

## Discussion

In the present study, we investigated for the first time the risk of healthcare workers with IBD to develop COVID-19 compared to control patients with IBD. In our cohort, the prevalence of COVID-19 was 8.7% of patients with IBD during year 2020. No difference was observed according to occupational status. We did not found any impact of IBD treatment on the risk of COVID-19. Lastly, we observed that patients with IBD were more likely to develop COVID-19 when their BMI was > 30 kg/m^2^.

Exposure to pathogen is a risk factor for opportunistic infection in the immunocompromised population [10]. Healthcare workers have higher susceptibility to respiratory infections in general and more specifically, zoonotic coronavirus outbreaks such as SARS, MERS and COVID-19 [11,12]. In a recent systematic review of 97 studies (all published in 2020) concerning 230,398 healthcare workers, the estimated prevalence of COVID-19 was 11% [[7], [8], [9], [10], [11], [12], [13], [14], [15]] using RT-PCR tests and 7% [[4], [5], [6], [7], [8], [9], [10], [11]] using serological tests while severe COVID-19 occurred in 5% [[3], [4], [5], [6], [7], [8]] of healthcare workers [5]. Being at the frontline response to COVID-19 results thus at higher risk of acquiring the disease. As an example, 2,600,498 patients were diagnosed with COVID-19 in France through 31 December 2020, accounting for 3.9% of the whole French general population [13]. In our study, the prevalence of COVID-19 was 9.8% which is similar to the latter data of patients without IBD. The distribution of COVID-19 was homogeneous across different healthcare workers' categories.

Surprisingly, there was no difference between healthcare workers with IBD (9.8%) and controls with IBD (7.7%, *P* = 0.41). France has been hit early during the European SARS-CoV-2 outbreak, early set-up of protective measure during year 2020 may explain such finding [14]. In the present study, the only healthcare workers' category who were at higher risk of COVID-19 was nurses' aides (14.0% vs. 5.5%, *P* = 0.02). We speculate that the closest contact with in-hospital patients that is associated with the work of nurses' aides may explain this difference. Independently of occupational status, we found an increased risk for our control population with IBD. In a recent systematic reviews with meta-analysis including 23 studies and 51,463 patients with IBD, through October, the prevalence of SARS-CoV-2 infection was 1.01% [0.92–1.10] [15]. It is conceivable that such results may reveal heterogeneity in the individual risk of getting COVID-19 for patients with IBD according to epidemiological differences of the COVID-19 outbreak and the anti-COVID-19 measures across countries and differences in the access to microbiological diagnosis using RT-PCR and/or serology. Notably, there was no difference between groups considering rates of intravenous biologics (infliximab and vedolizumab) administered at hospital infusion centre (74 (22.7%) in both groups, *P* = 1.00).

In the recent meta-analysis on the risk of COVID-19 in patients with IBD, 9 out of 23 studies reported specific outcomes according to ongoing treatments. Worse outcomes were noted in patients treated with steroids and aminosalicylates and better outcomes for those treated with biologics and immunosuppressants [15]. This could may be result from a potential effect of biologics and immunosuppressant on SARS-CoV-2 infection which has been demonstrated with tofacitinib and tocilizumab [16,17]. We did not observe such differences in our cohort. However, we have the relatively low number of patients did not allow us to performed subgroup analysis according to various IBD treatments.

In the present study, the only predictor of COVID-19 was obesity, defined as a BMI > 30 kg/m^2^. Obesity has been repeatedly reported as a risk factor of COVID-19 and severe COVID-19 in various cohort and epidemiological studies [[18], [19], [20], [21], [22]] [[18], [19], [20], [21], [22]]. The link between obesity and worse COVID-19 outcomes is complex including dysregulated immune response and altered mechanics of lungs and chest wall. Obesity is more and more reported among patients with IBD, accounting for approximately 15–40% of adults with IBD in cross-sectional studies [23,24]. Those considerations are not specific of SARS-CoV-2 but has also been previously pointed out for influenza virus and other respiratory viruses [25]. However, SARS-CoV-2 exerts an even higher cytokine storm in patients with underlying metabolic syndrome, diabetes and obesity [21].

Whereas, we included a large number of healthcare workers with IBD, we acknowledge a number of limitations in this study. First, the size of the present study may be too small to assess statistical difference between both groups. Second, data collection was retrospective during year 2021. However, all the participating centres are tertiary care centre with standardized clinical, biological, endoscopic and morphological prospective follow-up that lower the impact of such bias. Third, we focused on COVID-19 defined as symptomatic SARS-CoV-2 infection with positive RT-PCR test for SARS-CoV-2 on nasopharyngeal swab and/or a positive serological test for SARS-CoV-2 without prior vaccination. Nonetheless, this bias should be balanced in both groups. Fourth, we lost one third of the original cohort in both the healthcare worker and control groups and had to subsequently proceed to a rematch process of cases and controls. However, both groups were ultimately well balanced with few significant differences.

We concluded that healthcare workers with IBD did not exhibit an increased risk of COVID-19 compared with controls. Special attention should be given to nurses' aides with regard to closest contact with patients and underlying risk of transmission of SARS-CoV-2. Those data are reassuring as well as the low incidence of severe COVID-19 either in healthcare workers and control patients with IBD.

## Credit author statement

Conception and design of the study: CG, SN, LPB, PS, AA

Generation, Collection, Assembly, Analysis and/or Interpretation of data: AA, AB, PS, JMG, OD, SN, RA, VA, DL, CR, AB, MN, SV, LV, CS, PM, GB, AA

Drafting or revision of the manuscript: AA, AB, PS, JMG, OD, SN, RA, VA, DL, CR, AB, MN, SV, LV, CS, PM, GB, AA

Approval of the final version of the manuscript: AA, AB, PS, JMG, OD, SN, RA, VA, DL, CR, AB, MN, SV, LV, CS, PM, GB, AA

We wish to confirm that all the authors have approved the current submission.

## Conflicts of interest

Aurelien Amiot received consulting fees from Abbvie, Hospira, Janssen, Tillotts, Pfizer, Takeda, Gilead and Biocodex as well as lecture fees and travel accommodations from Abbvie, Janssen, Biocodex, Hospira, Ferring, Pfizer, Biogen, Amgen, Fresenius Kabi, Ferring, Tillotts, Takeda and MSD. This author also received advisory board fees from Gilead, Tillotts, Takeda and Abbvie

Jean-Marc Gornet received fees from Sanofi, Merck Serono, Roche, Novartis, Amgen and travel accommodation from Abbvie and MSD.

Olivier DeWit received consulting, lecture fees or travel accommodations from Abbvie, Biogen, Bristol Myers Squibb, Celltrion, Ferring, Fresenius Kabi, Galapagos, Janssen, MSD, Mylan, Pfizer, Sandoz.

Stephane Nancey received consulting fees from Merck, Abbvie, Takeda, Ferring, Norgine, Vifor Pharma, Novartis, Janssen-Cilag, Hospira, Takeda and HAC-Pharma.

Romain Altwegg received board or lectures fees from Abbvie, Janssen, Pfizer, Takeda, Amgen, Celltrion, Norgine, Ferring

Vered Abitbol received lecture fees from Biogen Amgen Sandoz Mylan Pfizer Takeda Janssen Gilead Tillots

David Laharie received counseling, boards or transports fees from Abbvie, Biogaran, Biogen, Ferring, HAC-pharma, Janssen, MSD, Novartis, Pfizer, Prometheus, Roche, Takeda, Theradiag, Tillots.

Catherine Reenaers received lecture fees from Abbvie, Takeda, Ferring, Pfizer, Galapagos, Celltrion, Janssen, Fresenius-Kabi, Bristol Myers Squibb, Thermo-Fisher and consultancy fees from Galapagos, Celltrion, Janssen, Fresenius-Kabi, Bristol Myers Squibb

Charlotte Gagniere received travel accommodations from Takeda.

Anthony Buisson has received research funding from Pfizer, lecture fees from Abbvie, Ferring, Hospira, MSD, Janssen, Sanofi-Aventis, Takeda and Vifor Pharma and consulting fees from Abbvie, Biogen, Janssen, Pfizer and Takeda.

Maria Nachury received board membership, consultancy, or lecture fees from Abbvie, Adacyte, Amgen, Arena, Biogen, CTMA, Celltrion, Ferring, Fresenius-Kabi, Janssen, Mayoli-Spindler, MSD, Pfizer, Takeda

Stephanie Viennot has received consulting fees from Abbvie, MSD, Takeda, Vifor Pharma and Ferring.

Lucine Vuitton received lecture fees from Abbvie, MSD, Takeda, Ferring, Mayoli, Janssen and Pfizer, and research grants from MSD, Takeda and Pfizer.

Guillaume Bouguen received lecture fees from Abbvie, Ferring, MSD, Takeda and Pfizer and consultant fees from Takeda, Janssen, Sandoz and Mylan.

Seksik P reports consulting fees from Pfizer, Astellas, Janssen, Fresenius Kabi, Takeda, Pilège and Biocodex and grants from Biocodex and Janssen.

These conflicts of interest are unrelated to the current work.

None for the remaining authors.

## Study funding

None

## Appendix

GETAID-INFOPRO study group All the members of the GETAID-INFOPRO study group are listed below: Aurelien Amiot, Charlotte Gagniere, Mathieu Uzzan, Department of Gastroenterology, Henri Mondor University Hospital, APHP, EC2M3-Equipe Universitaire, Paris Est-Créteil (UPEC) Val de Marne University, Creteil, France Anne Bourrier, Philippe Seksik, Laurent Beaugerie, Julien Kirchgesner, Harry Sokol, Cecilia Landman, Anne Bourrier, Sorbonne Université, École normale supérieure, PSL Research University, CNRS, INSERM, AP-HP, Department of Gastroenterology, Saint-Antoine Hospital, Laboratoire de biomolécules, LBM, F75005 Paris, France Matthieu Allez, Jean-Marc Gornet, Clautilde Baudry, Department of Gastroenterology, Saint Louis University Hospital, APHP, Paris, France Olivier DeWit, Department of HepatoGastroenterology, Catholic University of Leuven, University Hospital of Saint-Luc, Brussels, Belgium. Stephane Nancey, Gilles Boschetti, Bernard Flourié, Department of Gastroenterology, Hospices Civils de Lyon and Claude Bernard Lyon 1 University, Pierre-Benite, France Romain Altwegg, Department of Gastroenterology, Hôpital Saint-Eloi, University Hospital of Montpellier, Montpellier, France Vered Abitbol, Antoine Assaf, Department of Gastroenterology, Cochin University Hospital, University Paris 5 Descartes, Paris, France David Laharie, Florian Poullenot, Pauline Riviere, Department of HepatoGastroenterology, University Hospital of Bordeaux, Hôpital Haut-Lévêque, Bordeaux, France Catherine Reenaers, Edouard Louis, Department of Gastroenterology, University Hospital of Liège, Liège, Belgium. Anthony Buisson, Department of Hepato-Gastroenterology, University Hospital Estaing of Clermont-Ferrand, Université d'Auvergne, Clermont-Ferrand, France Maria Nachury, Pauline Wils, Department of Gastroenterology, Huriez University Hospital, Université Lille Nord de France, Lille, France Stephanie Viennot, Department of Gastroenterology, Caen University Hospital, F-14000, Caen, France Lucine Vuitton, Stephane Koch, Department of Gastroenterology, Besançon University Hospital, Besançon, France Carmen Stefanescu, Yoram Bouhnik, Xavier Treton, Department of Gastroenterology, IBD and Nutrition Support, Beaujon Hospital, University Paris 7 Denis Diderot, Clichy, France Philippe Marteau, Sorbonne Université-APHP, Tenon Hospital, Paris, France Guillaume Bouguen, Department of Gastroenterology, Pontchaillou Hospital and Rennes University, Rennes, France.
