# Supplementary material for: Risk of SARS-CoV-2 infection in healthcare workers with inflammatory bowel disease: a case-control study
Source: Infect Prev Pract. 2022 Dec 30;5(1):100267. doi: 10.1016/j.infpip.2022.100267 (PMC9800326; doi:10.1016/j.infpip.2022.100267)
Supplement: Multimedia component 1 [file mmc1.docx]

Table S1: Occupation characteristics of 326 healthcare workers with inflammatory bowel disease.

| **Occupation group** | **Number of healthcare workers with inflammatory bowel disease**  **(n = 482)** |
| --- | --- |
| **Physicians** | 83 (25.5%) |
| **Nurses** | 115 (35.3%) |
| **Nurses’ aides** | 43 (13.2%) |
| **Other healthcare workers**  Midwifes  Physiotherapists  Hospital psychologists  Hospital childcare workers  Dentists  Specialized nurses  Hospital pharmacists  Hospital social workers  Medical administrative assistants | 85 (30.1%)  9  14  6  8  6  6  18  6  14 |

Specialized nurse included nurse anesthesist, operating room nurse, nurse practitioner, nurse dietetitian and chief nurse.
